# Interictal EEG spikes increase perfusion in low-grade epilepsy-associated tumors: a pediatric arterial spin labeling study

**DOI:** 10.1007/s11547-024-01923-7

**Published:** 2024-11-12

**Authors:** Antonio Giulio Gennari, Giulio Bicciato, Santo Pietro Lo Biundo, Raimund Kottke, Dorottya Cserpan, Ruth Tuura O’Gorman, Georgia Ramantani

**Affiliations:** 1 Department of Neuropediatrics, University Children’s Hospital Zurich, Lenggstrasse 30, 8008 Zurich, Switzerland; 2 MR-Research Centre, University Children’s Hospital Zurich, Lenggstrasse 30, 8008 Zurich, Switzerland; 3 Department of Neurology, University Hospital Zurich, Rämistrasse 100, 8091 Zurich, Switzerland; 4 Department of Radiology, University Children’s Hospital Zurich, Lenggstrasse 30, 8008 Zurich, Switzerland; 5 University of Zurich, Zurich, Switzerland; 6 Children’s Research Centre, University Children’s Hospital Zurich, Zurich, Switzerland

**Keywords:** Pediatric epilepsy, Arterial spin labeling, Low-grade epilepsy-associated tumors, Sedation effect, Epileptogenicity

## Abstract

**Purpose:**

Arterial spin labeling (ASL), a noninvasive magnetic resonance (MRI) perfusion sequence, holds promise in the presurgical evaluation of pediatric lesional epilepsy patients, including those with low-grade epilepsy-associated tumors (LEATs). The interpretation of ASL-derived perfusion patterns, however, presents challenges. Our study aims to elucidate these perfusion changes in children with LEATs, exploring their correlations with clinical, electroencephalography (EEG), and anatomical MRI findings.

**Material and Methods:**

Our cohort included 15 children with LEAT-associated focal lesional epilepsy who underwent single-delay pseudo-continuous ASL imaging; eight were imaged under sedation. We assessed perfusion images both qualitatively and quantitatively, focusing on LEAT-related perfusion changes, as indicated by the asymmetry index (AI) and regional cerebral blood flow (rCBF).

**Results:**

ASL revealed LEAT-related perfusion changes in all but two patients: 12 LEATs were hypoperfused and one was hyperperfused relative to the contralateral brain parenchyma (CBP). LEATs showed significantly lower perfusion compared to CBP (median: 38.7 vs. 59.1 mL/100 g/min for LEAT and CBP, respectively; p value = 0.004, Wilcoxon–Mann–Whitney), regardless of sedation. Notably, elevated AI and rCBF values correlated with interictal spikes on EEG (median: -0.008 and 0.84 vs -0.27 and 0.58, respectively), but not to other clinical, EEG, or MRI variables (p value = 0.036, Wilcoxon–Mann–Whitney).

**Conclusions:**

By highlighting the connection between LEAT and brain perfusion, and by correlating perfusion characteristics and epileptogenicity, our research enhanced our understanding of pediatric epilepsy associated with LEATs. Also, by proving the robustness of these findings to sedation we confirmed the importance of adding ASL to epilepsy protocols to as a valuable tool to supplement anatomical imaging.

**Supplementary Information:**

The online version contains supplementary material available at 10.1007/s11547-024-01923-7.

## Introduction

Pharmacoresistance affects one-fourth of children with epilepsy^1^, and the presence of a brain lesion is one of its key determinants^2^. In carefully selected children with focal lesional epilepsy, epilepsy surgery is a safe and effective treatment option, offering the potential for seizure freedom without anti-seizure medication (ASM)^3^, thus positively impacting cognitive development^4^–^6^. Low-grade epilepsy-associated tumors (LEAT) are the second most common substrate of pediatric focal lesional epilepsy^7^ that carries the highest chances of postsurgical seizure freedom^8^–^10^. Presurgical evaluation seeks to define the epileptogenic zone (EZ), determine the lesion primarily responsible for seizure generation, and locate eloquent functional regions^3^. Magnetic resonance imaging (MRI) plays a central role in presurgical evaluation, guiding decisions for further exploration and eventual epilepsy surgery^11^. However, the delineation of the lesion, the EZ, and the understanding of their interrelations remain challenging, since even MRI-detectable lesions, like LEAT, may differ in terms of their epileptogenicity^12^. Therefore, auxiliary techniques may be necessary, such as nuclear medicine ones, which require the intravenous injection of radioactive tracers, exposure to radiation, and additional sedation sessions in younger ages, or invasive electroencephalography (EEG), which entails risks of hemorrhage, infection, and novel neurological deficit. To update presurgical assessment, boost surgical planning, and optimize postsurgical outcomes, novel noninvasive approaches are of uttermost importance.

Arterial spin labeling (ASL) is an MRI sequence that noninvasively images brain perfusion by magnetically labeling water protons in arterial blood^13^. ASL holds promise for the presurgical evaluation of children with focal lesional epilepsy^14^, ^15^. Its wide availability and recent standardization^16^ have boosted its use in this setting. However, while ASL aims to identify the EZ by highlighting abnormal perfusion patterns^14^, ^15^, ^17^, the distinct ASL patterns across various surgically treatable etiologies are not well-defined^18^, casting doubts on its reliability, utility, and relevance^19^. Specifically, in LEATs, our understanding of perfusion changes captured by ASL mainly derives from smaller subsets within larger lesional cohorts focused on differentiating between low- (LGG) and high-grade (HGG) gliomas via ASL perfusion metrics^20^–^26^ or comparing ASL with other presurgical investigations^14^, ^20^ across varying magnetic field strengths and ASL protocols^14^, ^20^–^26^. However, most studies have not adequately differentiated between pediatric and adult cases^22^, ^23^, ^26^, infratentorial and supratentorial lesions^21^, ^24^, ^27^, or diverse tumor types^25^–^27^. Moreover, there is lack of information regarding the epilepsy characteristics of LEAT cases, limiting the possibility to explore the connection between perfusion patterns encountered in LEATs and their correlations with clinical, EEG, and MRI features. Although LGG are generally hypoperfused, presumably due to their lower blood vessel density compared with HGG^21^, ^25^, ^28^, cases of hyperperfused LEATs have been noted without clear explanations^20^, ^22^, ^24^, ^29^, ^30^. Given that ASL-measured brain perfusion may also be impacted by age and sedation, understanding these patterns is crucial for leveraging ASL in diagnosing young children with LEAT^10^, ^31^–^34^, potentially minimizing the need for more invasive diagnostic methods.

Our study aimed to evaluate perfusion changes captured by ASL in children with LEAT, investigating their clinical, EEG, and anatomical MRI determinants. We qualitatively and quantitatively assessed perfusion images derived from a single-delay pseudo-continuous ASL sequence, identifying regional perfusion asymmetries and characterized them using quantitative values derived from cerebral blood flow (CBF) maps.

## Material and methods

### Patient selection

Patient data collection and analysis were conducted with approval from the local ethics committee (KEK-ZH 2024–00298), following established guidelines and regulations. Written informed consent for the reuse of clinical data for research purposes was obtained from all patients and their parents/legal guardians.

**Fig. 1:**
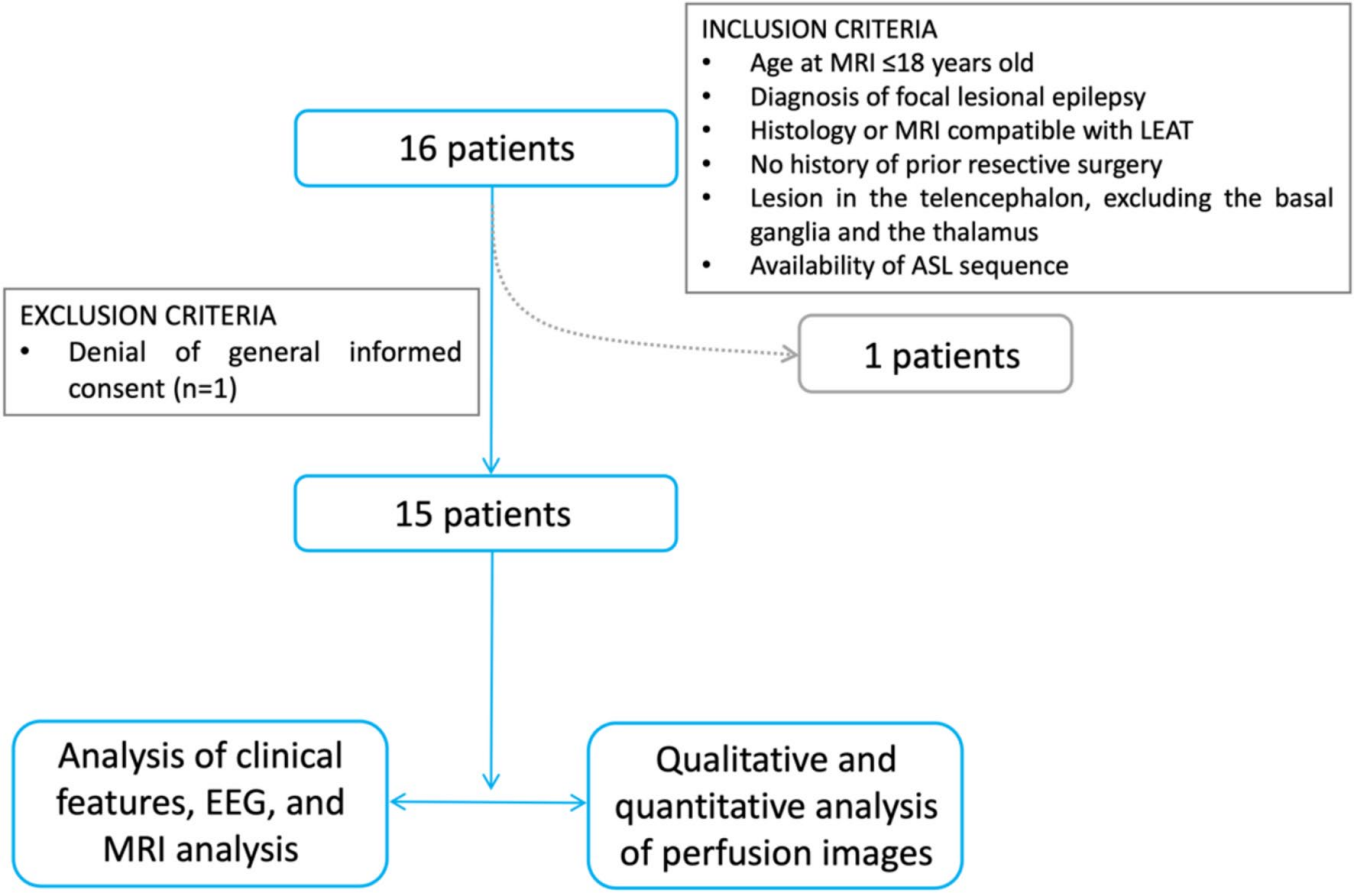
Patient and data selection

We searched our institutional database for children who had undergone brain MRI scans between November 1st, 2019, and July 1st, 2023, following our specialized epilepsy protocol^35^–^38^. We included patients who met the following criteria: 1) aged ≤ 18 years at the time of MRI, 2) diagnosed with focal structural epilepsy based on seizure semiology, EEG, and MRI findings, 3) diagnosed with a LEAT^39^, ^40^ based on radiological criteria^41^, confirmed by histopathology in surgical cases, 4) had not undergone resective epilepsy surgery at the time of inclusion, 5) had a lesion in the thelencephalon, not involving the basal ganglia or the thalamus, and 6) had ASL MRI sequence. We excluded 1) patients who did not provide general informed consent, and 2) MRI scans of insufficient quality for analysis (Fig. 1).

Details on the tumor classification used in this study and LEAT selection criteria are provided in the** Supplementary material.**

### EEG acquisition and analysis

We considered the EEG recording closest in time to each MRI scan for analysis^17^. Details on EEG acquisition technique and evaluation can be found in the **Supplementary material**. We documented the presence of focal slowing and spikes, previously linked to ASL perfusion changes^17^, ^42^, ^43^. Spike frequency was calculated during the first hour of wakefulness and, when applicable, the first hour of non-rapid eye movement (NREM) sleep^44^. Spike frequency was subdivided into two categories^45^, ^46^: “frequent spikes” (≥ 60) and “non-frequent spikes” (< 60) based on the number of spikes per hour.

### MRI acquisition

All MRI scans were performed using a 3T scanner (Discovery MR 750 or Signa Premium, GE Medical Systems), equipped with an 8-channel coil^35^, ^37^, ^38^. Both anatomical and perfusion images were obtained for all cases. Detailed image acquisition parameters can be found in our previous studies^17^, ^36^. Quantitative perfusion maps were automatically generated using GE reconstruction software. Sedation was administered for MRI acquisition if clinically indicated^15^. Since no clinical seizures were observed during the MRI scans, the perfusion patterns depicted in the images were interpreted as reflective of brain perfusion during the interictal state.

### MRI qualitative analysis

**Fig. 2:**
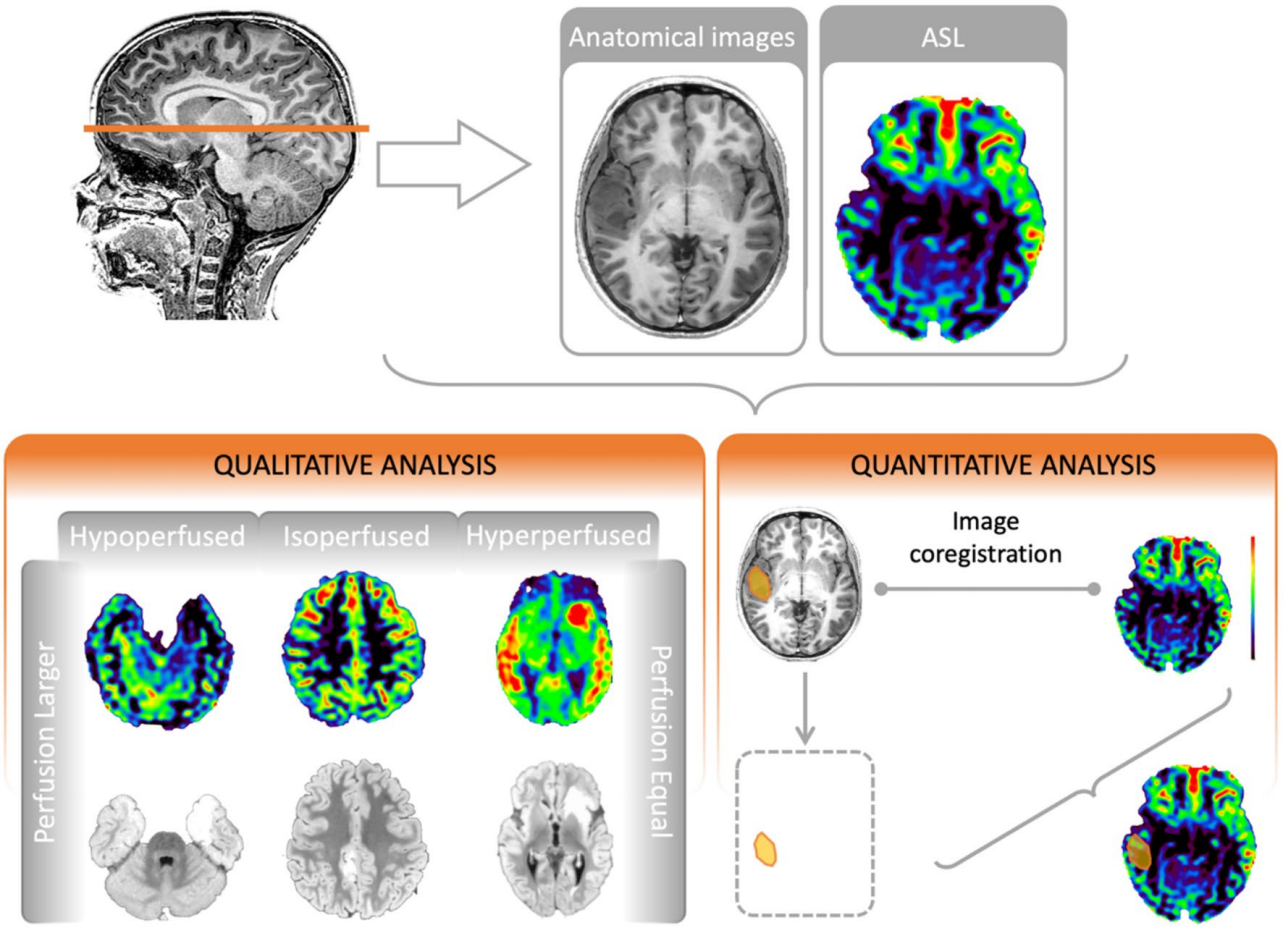
**Image processing pipeline.** MRI analysis pipeline. Qualitative analysis: Perfusion changes related to low-grade epilepsy-associated tumors (LEAT) were classified as hyperperfused, isoperfused, or hypoperfused compared to the contralateral brain parenchyma (CBP). Furthermore, the extent of perfusion changes associated with LEAT was dichotomized as larger or equal as compared to the lesion extent on anatomical images. Quantitative analysis: A volumetric binary label was created based on the appearance of LEAT on anatomical images. Additionally, ASL images were co-registered with the anatomical images, extending the coregistration to binary labels. This allowed the quantification of the mean cerebral blood flow (CBF) value within the binary label representing the LEAT, as well as that of the CBP after flipping the label to the contralateral side. CBF: cerebral blood flow; CBP: contralateral brain parenchyma; LEAT: low-grade epilepsy-associated tumor

The MRI qualitative analysis followed the methodology outlined in our previous study^17^ (Fig. 2) or briefly summarized in the **Supplementary material**.

### MRI quantitative analysis

Quantitative analysis aimed to quantify the perfusion values in the LEAT and contralateral brain parenchyma (CBP) using Slicer (https://www.slicer.org) and FSL (www. fmrib.ox.ac.uk/fsl). The post-processing pipeline followed the steps outlined in our previous study^17^**(**Fig. 2**)**. To normalize the automatically generated CBF values for each patient and facilitate the quantification of perfusion asymmetries between cerebral hemispheres, we utilized the asymmetry index (AI)^14^, ^19^, ^47^ and relative cerebral blood flow (rCBF)^21^, ^24^, ^25^, ^27^–^29^. The AI was calculated using the following formula:

$$AI= \frac{CB{F}_{LEAT}-CB{F}_{CBP}}{CB{F}_{LEAT}+CB{F}_{CBP}},$$

whereas the rCBF was calculated using the following formula:

$$\text{rCBF}= \frac{CB{F}_{LEAT}}{CB{F}_{CBP}}$$

In both approaches, we calculated the CBF of the LEAT and its homotopic CBP by manually outlining volumetric regions of interest (ROI) encompassing the entire LEAT. For rCBF, we favored this approach over placing bidimensional ROI on areas of the LEAT with the highest perfusion values based on visual inspection. Our approach was guided by: 1) the limited correlation between visually and automatically calculated maximum LEAT CBF values^48^, and 2) the hypoperfused pattern observed in most LGGs^23^, which makes visually identifying regions with the highest perfusion challenging and may compromise the reproducibility of results. Additionally, using mean values helps reduce data variability, thereby strengthening potential associations.

### Statistical analysis

Dichotomous and categorical variables were reported using absolute numbers and percentages. Continuous variables were described using either median and inter-quartile range (IQR) or mean ± standard deviation (SD). Data distribution was assessed using the Shapiro–Wilk test. The distribution of categorical variables was assessed using the Chi-Square and Fisher’s exact tests. Given the small sample size, group-wise comparisons of continuous variables were conducted using two-tailed nonparametric tests. The correlation between variables was assessed using Spearman’s correlation coefficient.

Bland–Altman plots were used in a sensitivity analysis conducted to assess the reliability of the automatically generated CBF values used in this study. Given the close relation between ASL signal and blood T1 relaxation time, which in turn is directly associated with blood hematocrit (Hct)^49^, corrected CBF values were calculated using the results of blood tests conducted within 2 months from the MRI or using normative Hct values corresponding to the age at MRI in all other cases. These corrected values were then compared with the automatically generated CBF values.

The statistical analysis was conducted using *R* software (https://www.r-project.org) version *4.0.5*, with statistical significance set at *p value* ≤ 0.05.

## Clinical features

Our study included fifteen children (67% female, Fig. 1). The median age at epilepsy onset was 6 years (IQR: 1.5–9), the median age at MRI was 8.2 years (IQR: 2.4–13.6), and the median epilepsy duration was 1.8 years (IQR: 0.4–3.8, Table 1). Three (20%) children had been seizure free for a year, 4 (27%) had < 30, and 8 (53%) had ≥ 30 seizures per month. Eleven (73%) children underwent epilepsy surgery, and one (7%) underwent brain biopsy. The most common histopathological diagnoses were: ganglioglioma, desmoplastic infantile ganglioglioma, and pilocytic astrocytoma, totaling 40% of cases (Table 1). All tumor specimens, including the biopsy, underwent immunohistochemical assessment, and nine (82%) underwent next-generation sequencing. None of the specimens tested positive for IDH-1 r132h or H3K27me3. However, BRAF v600E and ATRX mutations were identified in three (17%) and two (25%) cases, respectively. All specimens exhibited a low mitotic index, as assessed by MIB-1 or Ki-67 staining.

**Table 1:** Clinical features, EEG and MRI findings, and histopathology

**Clinical features**			**n = 15**
	Male, n (%)		5 (33%)
	Age at MRI in y, median (IQR)		8.2 (2.4 to 13.5)
	Age at epilepsy onset in y, median (IQR)		6 (1.5 to 9.0)
	Epilepsy duration in y, median (IQR)		1.8 (0.4 to 3.8)
	Seizure frequency	Seizure free	3 (20%)
		< 30 seizures	4 (27%)
		≥ 30 seizures	8 (53%)
	Sedation for MRI acquisition, n (%)		8 (53%)
	ASM at MRI, n (%)	No ASM	2 (13%)
		One ASM	6 (40%)
		Two or more ASMs	7 (47%)
**EEG findings**	Focal slowing, n (%)		8 (53%)
	Spikes, n (%)		9 (60%)
	Frequent spikes, n (%)		2 (13%)
**MRI findings**	Right lateralization, n (%)		8 (53%)
	Lobar localization, n (%)	Frontal	3 (20%)
		Temporal	7 (47%)
		Posterior	3 (20%)
		Multilobar	2 (13%)
	Lesion volume in mm^3^, median (IQR)		11,834 (3984 to 23,124)
**Perfusion images**			
*Perfusion patterns*	Isoperfused, n (%)		2 (13%)
	Hypoperfused, n (%)		12 (80%)
	Hyperperfused, n (%)		1 (7%)
*Perfusion extent*	Larger, n (%)		5 (38%)
	Equal, n (%)		8 (62%)
**Histopathology (n = 12)**	Age at surgery/biopsy, median (IQR)		11.4 (4.1 to 13.5)
	Histology, n (%)	Ganglioglioma	2
		Desmoplastic infantile ganglioglioma	2
		Pilocytic astrocytoma	2
		Glioneuronal tumor	1
		Diffuse astrocytoma	1
		Angiocentric glioma	1
		LGG/Glioneuronal tumor	1
		LGG	1
		Pleomorphic xanthoastrocytoma	1

ASM, anti-seizure medication; IQR, inter-quartile range; LGG, low-grade glioma; n, number; Posterior: involving either the parietal or the occipital lobe; y: years. Lobar involvement was classified as frontal, temporal, posterior, and multilobar. Lesions located in the parietal or occipital lobe were grouped as posterior, while lesions defined as multilobar involved more than one lobe, irrespective of the lobes involved

We analyzed 15 MRIs (53% under sedation) and the corresponding EEGs (3 during wakefulness; 12 during both wakefulness and sleep). The median interval between MRI and EEG was ± 6 days (IQR: 3–18). Nine (60%) EEGs were obtained within a week of MRI, and three (20%) on the same day (**Supplementary material**). Two MRI scans (13%) were conducted without any ASM, while the remaining 13 (87%) were performed with one to four ASMs (Table 1 and **Supplementary material**). Levetiracetam was the most commonly used ASM (nine cases, 60%), either alone or in combination with other medications (**Supplementary material**). None of the MRIs or EEGs were acquired under a ketogenic diet. Additionally, eight of 15 MRI scans (53%) were conducted under sedation, with propofol being the most frequently administered sedative (seven cases, 88%), either in mono- or polytherapy (Table 1 and **Supplementary material**).

### EEG characteristics

The median EEG duration was 60 min (IQR: 35–5040 min); 10 (67%) children underwent EEGs lasting ≥ 24 h. Focal slowing was observed in 8 (53%) EEGs, while spikes were observed in 9 (60%) EEGs (Table 1).

### MRI qualitative analysis

Eight (53%) LEATs located the right hemisphere. Seven LEATs were situated in the temporal lobe, three in the frontal lobe, three in posterior regions, and two were multilobar (fronto-insular; fronto-temporal) (Table 2). LEAT-associated perfusion changes were observed in 13 (87%) cases: 12 were hypoperfused, while one was hyperperfused. Temporal and extratemporal LEATs showed ASL perfusion changes in equal rates (86% each). In five cases, perfusion changes extended beyond the boundaries of the LEAT observed on anatomical images, while in eight cases, the perfusion changes matched the extent of the LEAT. Additionally, two cases showed an isoperfused pattern. A hypoperfused pattern was observed in seven out of eight (88%) scans acquired under sedation and in five of seven (71%) scans performed without sedation (**Supplementary material**). The two cases characterized by frequent spikes (a ganglioglioma and an angiocentric glioma) showed a hyperperfused and an isoperfused pattern, respectively. Pilocytic astrocytomas (two cases) and desmoplastic infantile gangliogliomas (two cases) were hypoperfused. Conversely, the two ganglioglioma cases showed a hyperperfused and an isoperfused pattern, respectively.

**Table 2:** Comparison between quantitative ASL findings and clinical, EEG, and MRI features

Clinical, EEG, and MRI features		Asymmetry index	*p* value	*Relative CBF*	*p* value
Age at MRI in y^$^		− 0.05	0.86	− 0.05	0.86
Age at epilepsy onset in y^$^		0.07	0.74	0.06	0.82
Epilepsy duration in y^$^		− 0.06	0.83	− 0.06	0.83
Seizure frequency, median (IQR)^#^	< 30 monthly	− 0.25 (− 0.27 to -0.12)	0.23	0.60 (0.57 to 0.79)	0.23
	≥ 30 monthly	− 0.11 (− 0.22 to -0.02)		0.82 (0.64 to 0.96)	
Presence of focal slowing, median (IQR)^#^	Positive	− 0.11 (− 0.26 to − 0.03)	0.54	0.81 (0.60 to 0.95)	0.54
	Negative	− 0.20 (− 0.27 to − 0.14)		0.66 (0.58 to 0.76)	
Presence of spikes, median (IQR)^#^	Positive	− 0.08 (− 0.17 to − 0.03)	0.036*	0.84 (0.71 to 0.95)	0.036*
	Negative	− 0.27 (− 0.27 to − 0.26)		0.58 (0.57 to 0.59)	

IQR, inter-quartile range; LEAT, low-grade epilepsy-associated tumors; MRI, magnetic resonance; y, years; ^$^, Spearman’s correlation; ^#^, Mann–Whitney test; *statistical significance. The AI values are provided in relation to clinical features, EEG, and anatomical MRI findings

### MRI quantitative analysis

The median LEAT volume was 11,834 mm^3^ (IQR: 3984–23,124 mm^3^).

The median CBF was 38.7 mL/100 g/min (IQR: 32.9–55.3 mL/100 g/min) within the LEATs and 59.1 mL/100 g/min (IQR: 54.6–67.5 mL/100 g/min) within the CBP. These values differed significantly (*p value* = 0.004, Wilcoxon–Mann–Whitney), in line with the expected hypoperfusion in LEATs. CBF values in CBP were significantly lower under sedation (54.6 mL/100 g/min, IQR: 39.8–60.6 mL/100 g/min) than without sedation (66 mL/100 g/min, IQR: 60.2–70.3 mL/100 g/min; *p value* = 0.041, Wilcoxon–Mann–Whitney), whereas LEAT CBF values were not affected by sedation status (*p value* = 0.12, Wilcoxon–Mann–Whitney). The Htc-corrected CBF values of LEAT and CBP were within acceptable range (**Supplementary material**).

**Fig. 3:**
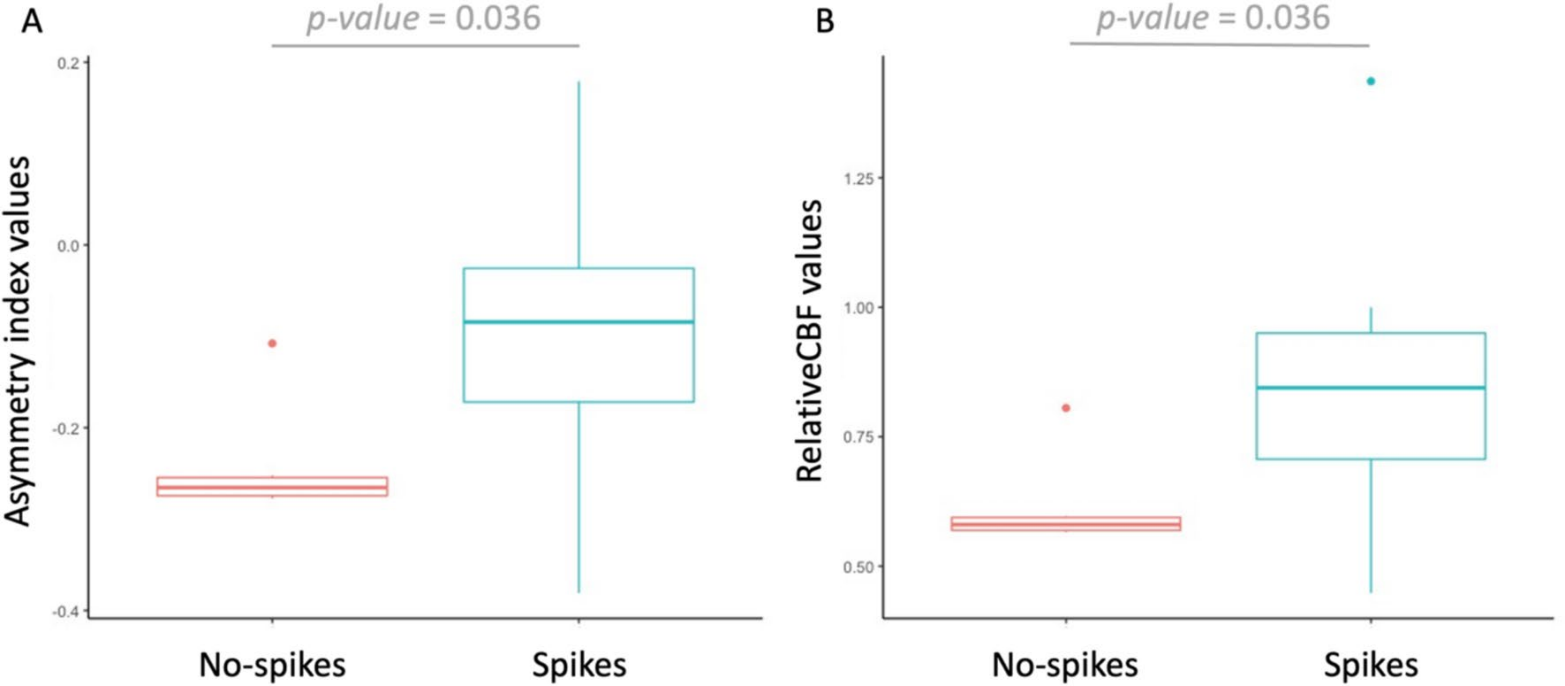
**Interictal spikes determined a higher asymmetry index (AI) and higher relative CBF (rCBF).** (A, B) The boxplots illustrate the correlation between spikes in interictal EEG and perfusion values. Both the AI and rCBF were higher in patients with spikes on interictal EEG (*p* values = 0.036, Wilcoxon–Mann–Whitney)

The mean AI was -0.15 ± 0.14, while the mean rCBF was 0.76 ± 0.25. Sedation had no impact on either AI or rCBF (both *p value* = 0.46, Wilcoxon–Mann–Whitney). Higher AI and rCBF values were associated with the presence of interictal spikes in EEG (both *p values* = 0.036, Wilcoxon–Mann–Whitney, Fig. 3). Of note, this association maintained its significance using AI and rCBF based on Htc-corrected values (both *p values* = 0.036, Wilcoxon–Mann–Whitney, **Supplementary material**). AI and rCBF values in the two cases with frequent spikes were 0.18, -0.17, 1.4, and 0.7, respectively; the median of cases with spikes were -0.08 (IQR: -0.16– -0.03) and 0.84 (IQR: 0.72–0.94), while that of cases with no spikes were -0.27 (IQR: -0.27– -0.26) and 0.58 (IQR: 0.57–0.59), respectively.

## Discussion

To our knowledge, this study is the first to explore perfusion changes captured by ASL in children with LEAT, focusing on identifying the determinants of these changes. Our findings highlight that ASL identified LEAT-related perfusion changes in the majority of our study cohort. Qualitative analysis demonstrated a predominantly hypoperfused pattern. Quantitative analysis showed that higher AI and rCBF values were linked to interictal spikes on EEG, regardless of sedation status. These results reinforce the growing body of evidence, supporting that ASL is a valuable tool in the presurgical evaluation of focal lesional epilepsies and underscore a link between lesion perfusion and its epileptogenic potential, particularly in pediatric patients.

### Most LEATs present perfusion changes on ASL, regardless of sedation status

In our study, encompassing a wide range of LEATs across both temporal and extratemporal regions, and thus comprehensively reflecting LEAT distribution^50^, ^51^, we found that all but two cases demonstrated LEAT-related perfusion changes, as captured by ASL. This observation is consistent with findings from other studies on focal cortical dysplasia (FCD)^17^. Particularly, we found that 86% of temporal and extratemporal LEATs in our pediatric cohort showed perfusion changes on ASL, in contrast to the 72% and 55% rates of metabolism changes observed in positron emission tomography scans of temporal and extratemporal lobe lesions in an adult cohort^52^. Importantly, ASL identified these LEAT-associated perfusion changes irrespective of sedation during imaging for half of our study cohort. This observation not only reaffirms our previous findings in children with FCD-related epilepsy^17^ but also applies our insights to another crucial epilepsy substrate suitable for surgical treatment, validating the utility and applicability of ASL for presurgical evaluation in younger children^10^, ^31^–^34^, particularly those requiring sedation for imaging studies. Accounting for sedation effects in perfusion imaging is crucial^53^ for precise interpretation and optimal clinical management. Our study addresses a previously unmet need in this area^25^ and suggests broader implications beyond the delineation of the epileptogenic lesion and the related EZ, extending to children with brain tumors where ASL discriminates between HGGs and LGGs^21^, ^25^. Previous pediatric tumor grading based on ASL either exclusively considered scans performed without sedation^21^ or did not consider sedation status^25^, ^27^. By affirming the reliability of ASL metrics regardless of sedation, our work enhances confidence in the clinical utility of ASL.

### LEATs are predominantly hypoperfused

In our study, hypoperfusion emerged as the dominant perfusion pattern among LEATs, consistent with research linking hypoperfusion with a reduced microvascular density, lower vessel turnover, and smaller vessel sizes in LGGs compared to HGGs^21^, ^29^. Interestingly, lesion location (temporal versus extratemporal) or depth (superficial versus deep) did not impact these perfusion patterns, consistent with existing pediatric brain tumor perfusion research^21^, ^29^. Moreover, the observed hypoperfusion in LEATs may reflect their lower epileptogenicity compared to FCDs, as indicated by an electrocorticography study^54^, in line with the differences observed between the hyperperfused active tubers and hypoperfused inactive tubers in tuberous sclerosis^55^, ^56^. Considering lesion epileptogenicity is crucial in ASL studies assessing patients with overt or suspected lesional epilepsy or leveraging perfusion measurements to estimate tumor grades. Our analysis also revealed single cases of LEATs exhibiting hyperperfusion or isoperfusion, echoing previous accounts of hyperperfused LGGs, including two pilocytic astrocytomas, a ganglioglioma, and a grade II astrocytoma^20^, ^22^, ^29^, ^30^. Interestingly, the high vascularity of pilocytic astrocytoma^57^, ^58^ challenges the presumed link between tumor vascularity and hypoperfusion, suggesting that variations in perfusion may also stem from differences in epileptic activity. Notably, our study uniquely highlighted that LEATs associated with more frequent epileptic spikes on interictal EEG were hyperperfused, introducing a new aspect not previously explored. We found no correlation between perfusion patterns and any epidemiological, epilepsy-related, or lesion-related features, consistent with our prior study on FCD^17^. This may be attributed to the complex nature of epilepsy, its diverse manifestations, and the intricate characteristics of seizure-generating lesions, complicating any straightforward analysis of how individual clinical factors impact imaging findings. Nonetheless, compared to FCD (44%)^17^, LEAT-related perfusion changes were commonly (62%) limited to the lesion extent on anatomical images, suggesting a more lesion-centered epileptic network.

### Higher ASL quantitative metrics associated with Interictal spikes on EEG

Higher AI and rCBF values were correlated with interictal spikes on EEG, marking a novel observation in the study of LEATs. This is the first study to link a perfusion increase to the epileptogenic activity of LEATs. Although most of these lesions predominantly displayed a hypoperfused pattern upon visual inspection, our findings indicate that AI and rCBF can provide diagnostic insights beyond those offered by more basic imaging analysis techniques. This aligns with recent research in temporal lobe epilepsy patients, which found increased aerobic glycolysis ipsilateral to the epileptic focus during the interictal state or reflect interictal fluctuations between quiescent and active states^59^, ^60^. Despite the methodological differences, our rCBF values were consistent with previous reports^21^. This similarity may be attributed to the small size of some LEATs or the presence of cystic components^61^ within others. Although the presence of cystic or hypovascular regions within a LEAT should theoretically lower overall perfusion values, this effect is averaged out by analyzing the entire CBP within the same label, thus including the typically hypovascular white matter, as in our study. Conversely, in smaller lesions, the influence of these components is likely minimal. Hence, our approach, by encompassing the whole lesion, may inadvertently compare the tumor area with the highest ASL signal to the contralateral gray matter, similar to other studies^21^, ^25^, ^29^. However, by assessing the entire lesion, our approach minimizes errors that could arise from subjective ROI placement.

### Future directions

Of note, LEAT CBF and AI values in LEATs were lower compared to those of FCD, as reported in our previous work^17^. This finding opens new avenues for future research, utilizing ASL in larger cohorts of children with focal lesional epilepsy related to FCD and LEAT. Future studies could address cases where presurgical MRI discrimination between these histological entities is particularly challenging and investigate whether coexisting pathologies, such as in the case of FCD type IIIb, exhibit distinct perfusion patterns or CBF-related values. This research could support the integration of ASL into clinical protocols, enhancing noninvasive presurgical evaluation, patient counseling, and prognostication. Larger studies may validate ASL as a reliable (and safer) alternative to positron emission tomography. Although our study did not link clinical features with LEAT-associated perfusion metrics, global brain perfusion may indirectly reflect brain maturation and disease state, potentially connecting to cognitive development^35^ or treatment efficacy^62^. Furthermore, the diagnostic potential of ASL may extend beyond pharmacoresistant lesional epilepsy to more common pediatric epilepsy syndromes, such as self-limited epilepsy with centrotemporal spikes, by identifying disease-specific imaging patterns.

## Conclusion

Our study demonstrates that most LEATs are associated with ASL perfusion changes, irrespective of sedation or lesion location. The prevalent perfusion pattern among LEATs was hypoperfusion, suggesting a characteristic trait of this structural etiology. Notably, higher AI and rCBF values correlated with the presence of epileptic spikes on interictal EEG. These observations reinforce the potential of ASL as a noninvasive imaging biomarker for children with focal lesional epilepsy and shed light on the perfusion characteristics of LEATs. Moreover, our findings confirm the association between interictal epileptic activity and brain perfusion, laying a solid groundwork for incorporating ASL into both clinical management and epilepsy research. Specifically, ASL stands out for its potential to improve lesion detection rates and facilitate lesion characterization, thereby minimizing reliance on more invasive imaging methods. Additionally, elucidating the relationship between neuronal activity and perfusion presents exciting opportunities to advance our understanding and theoretical models of epileptogenesis^63^.

## Supplementary Information

Below is the link to the electronic supplementary material.

Supplementary file1 (PDF 297 KB)
