# Supplementary material for: Interictal EEG spikes increase perfusion in low-grade epilepsy-associated tumors: a pediatric arterial spin labeling study
Source: Radiol Med. 2024 Nov 12;130(1):63–73. doi: 10.1007/s11547-024-01923-7 (PMC11882625; doi:10.1007/s11547-024-01923-7)
Supplement: Supplementary file 1 — Supplementary file1 (PDF 297 KB) [file 11547_2024_1923_MOESM1_ESM.pdf]

Supplementary material

TUMOR CLASSIFICATION AND LOW-GRADE EPILEPSY-ASSOCIATED (LEAT) TUMOR SELECTION CRITERIA . 2

ELECTROENCEPHALOGRAPHY (EEG) ACQUISITION TECHNIQUE AND EVALUATION 2

ARTERIAL SPIN LABELING IMAGES (ASL) QUALITATIVE ANALYSIS 2

SUPPLEMENTARY FIGURE 1. BLAND-ALTMANN PLOTS COMPARING AUTOMATICALLY GENERATED CEREBRAL BLOOD FLOW (CBF) VALUES TO HEMATOCRIT CORRECTED ONES IN LOW-GRADE EPILEPSY ASSOCIATED TUMORS (LEATS) AND CONTRALATERAL BRAIN PARENCHYMA (CBP). 3

SUPPLEMENTARY FIGURE 2. INTERICTAL SPIKES DETERMINED HIGHER HEMATOCRIT-CORRECTED ASYMMETRY INDEX (AI) AND RELATIVE CBF (RCBF) VALUES. 3

SUPPLEMENTARY TABLE 1. CLINICAL FEATURES, EEG AND MRI FINDINGS, NEED FOR SEDATION, AND ANESTHETICS USED FOR SEDATION. 4

SUPPLEMENTARY TABLE 2. THE PERFUSION PATTERNS INDUCED BY LOW-GRADE EPILEPSY ASSOCIATED TUMORS (LEAT) ON ARTERIAL SPIN LABELLING (ASL) IMAGES DID NOT CORRELATE WITH CLINICAL, EEG, OR MRI FINDINGS.. 5

SUPPLEMENTARY TABLE 3. PERFUSION CHANGES IN ARTERIAL SPIN LABELLING (ASL) LARGER THAN THE LOW-GRADE EPILEPSY ASSOCIATED TUMORS (LEAT) DID NOT CORRELATE WITH CLINICAL, EEG, OR MRI FINDINGS. 5

BIBLIOGRAPHY 5

Tumor classification and low-grade epilepsy-associated (LEAT) tumor selection criteria

We used the 2021 World Health Organization (WHO) Classification of Tumors of the Central Nervous System[1] for histological classification and grading of LEAT. LEAT definition was derived by Luyken *et al.*[2] and a subsequent revision[3]. Additionally, we included two other histopathological entities among LEAT: 1) desmoplastic infantile ganglioglioma, recently categorized within glioneuronal tumours[1] according to the 2021 WHO classification, and 2) diffuse astrocytoma, which shares characteristics such as WHO grade (I), mutation (MyB), and classification (pediatric type diffuse LGG), with angiocentric glioma[1], which has been recently included among LEAT[3].

Electroencephalography (EEG) acquisition technique and evaluation

EEGs were recorded using the Micromed® or Deltamed® systems, using 21 EEG electrodes positioned according to the international 10-20 system. EEGs were reviewed by a fully trained neurologist, blinded to the clinical report. EEGs during wakefulness and sleep were analyzed following the guidelines by the American Academy of Sleep Medicine[4] for sleep staging.

Arterial spin labeling images (ASL) qualitative analysis

Briefly, a fully trained radiologist with 8 years of experience in neuroradiology characterized LEAT lateralization, lobar, sub-lobar location, and depth based on anatomical images. Perfusion images were inspected for hemispheric disparities and correlated with anatomical images to identify regional perfusion changes corresponding to each LEAT. Perfusion changes were considered present if visible in at least two consecutive slices[5]. Lesion-associated perfusion changes were categorized as hypoperfused or hyperperfused based on their appearance relative to the contralateral brain parenchyma (CBP, **Fig. 2**). Cases with no perfusion changes were considered isoperfused. Furthermore, the extent of perfusion changes relative to the lesion extent on anatomical images was assessed and categorized as "equal" (indicating that the extent of perfusion changes matched the extent of the LEAT on anatomical images) or "larger" (indicating that the extent of perfusion changes exceeded the extent of the LEAT on anatomical images)[6].

Supplementary Figure 1. Bland-Altmann plots comparing automatically generated cerebral blood flow (CBF) values to hematocrit corrected ones in low-grade epilepsy associated tumors (LEATs) and contralateral brain parenchyma (CBP).

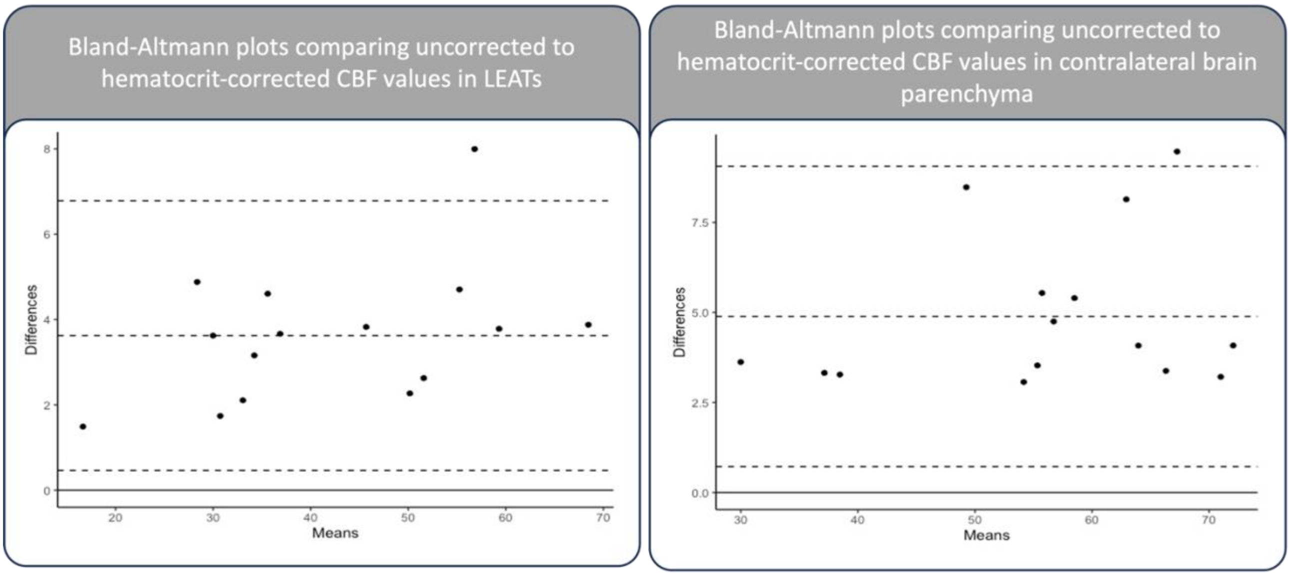

Agreement between cerebral blood flow (CBF) values and corrected hematocrit corrected CBF values.
(A, B) Bland-Altman plots comparing the automatedly calculated and the hematocrit-corrected CBF values in the low-grade epilepsy associated tumors (LEATs, A) and the contralateral brain parenchyma (CBP, D). Absolute difference values and confidence intervals were deemed within acceptable ranges.

Supplementary Figure 2. Interictal spikes determined higher hematocrit-corrected asymmetry index (AI) and relative CBF (rCBF) values.

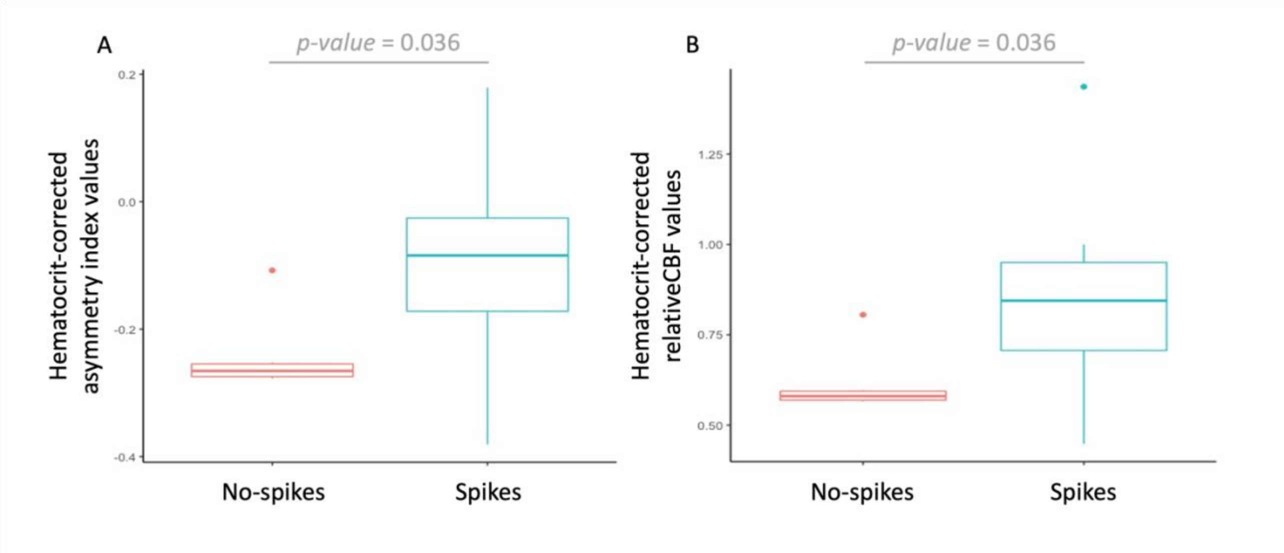

A, B) The boxplots illustrate the existing correlation between the presence of spikes in interictal EEG and perfusion values. The AI and rCBF generated by using hematocrit-corrected values were higher in patients having spikes on interictal EEG (p-values = 0.036, Wilcoxon-Mann-Whitney).

Supplementary Table 1. Clinical features, EEG and MRI findings, need for sedation, and anesthetics used for sedation.

Pat. Nr.	Sex	Age at epilepsy onset, y	Age at MRI, y	Seizure frequency, monthly	Focal slowing in EEG	Spikes in EEG	Frequent spikes in EEG	Sedation for MRI	Anaesthetics for sedation	Lobar localization	ASMs at scan	Lesion volume, mm^3	ASL perfusion pattern	ASL perfusion extent	Histology
1	F	2	2	>30 seiz.	Yes	Yes	Yes	Yes	Ket., Prop.	Multilobar	LEV, OXC, VPA	21751	Hyperperfused	Equal	Ganglioglioma
2	F	8	11	>30 seiz.	No	Yes	Yes	No	No sedation	Parietal	LEV, TPM	7164	Isoperfused	-	Angiocentric Glioma
3	M	7	8	>30 seiz.	No	Yes	No	Yes	Ket., Prop.	Temporal	LEV, OXC	5244	Hypoperfused	Equal	Pilocytic Astrocytoma
4	M	6	11	>30 seiz.	No	Yes	No	No	No sedation	Parietal	LEV, OXC	1977	Hypoperfused	Larger	LGG/Glioneuronal Tumor
5	M	0.1	2	≤30 seiz.	Yes	No	No	Yes	Mida., Fent.	Occipital	LEV	39138	Hypoperfused	Larger	-
6	F	1	8	seiz. free.	No	No	No	Yes	Prop.	Temporal	None	2350	Hypoperfused	Equal	-
7	F	0.7	0.8	>30 seiz.	Yes	Yes	No	Yes	Ket., Prop.	Temporal	CBZ, CLB	2724	Hypoperfused	Equal	Pilocytic Astrocytoma
8	F	17	19	>30 seiz.	No	No	No	No	No sedation	Frontal	LTG	14784	Hypoperfused	Equal	Glioneuronal Tumor
9	F	14	14	≤30 seiz.	Yes	Yes	No	No	No sedation	Temporal	None	24497	Hypoperfused	Equal	Pleomorphic Xanthoastrocytoma
10	F	2	23	≤30 seiz.	No	No	No	Yes	Ket., Prop.	Multilobar	LEV	10404	Hypoperfused	Larger	Desmoplastic Infantile Ganglioglioma
11	F	10	14	seiz. free.	No	No	No	No	No sedation	Frontal	LEV	87466	Hypoperfused	Equal	LGG
12	F	12	15	>30 seiz	Yes	Yes	No	No	No sedation	Temporal	LEV, CBZ	13940	Isoperfused	-	Ganglioglioma
13	M	6	6	>30 seiz	Yes	Yes	No	Yes	Ket., Prop.	Temporal	LEV, OXC	38106	Hypoperfused	Equal	Diffuse Astrocytoma
14	M	0.6	2	≤30 seiz.	Yes	Yes	No	Yes	Prop., Nalb.	Temporal	VPA	11834	Hypoperfused	Larger	Desmoplastic Infantile Ganglioglioma
15	F	6	14	seiz. free	Yes	No	No	No	No sedation	Frontal	LTG	820	Hypoperfused	Larger	-

Pat: patient; nr: number; M: male; F: female; y: years; seiz.: seizure; Ket.: ketamine; Prop.: propofol; Nlp: nalbuphine; Mida: midazolam; ASM: anti-seizure medication; VPA: valproate; TPM: topiramate; OXC: oxcarbazepine; VGB: vigabatrin; CORT: hydrocortisone; LEV: levetiracetam; CLB: clobazam; STM: sultiam; LTG: lamotrigine; CBZ: carbamazepine; ASL: arterial spin labelling;

Supplementary Table 2. The perfusion patterns induced by low-grade epilepsy associated tumors (LEAT) on arterial spin labelling (ASL) images did not correlate with clinical, EEG, or MRI findings.

The perfusion patterns depicted in ASL images were simplified by combining hyperperfused and isoperfused cases into a single class labelled as "other". These were analyzed in relation to clinical features, EEG, and anatomical MRI findings.

**CLINICAL, EEG, AND MRI FEATURES**	**ASL PERFUSION EXTENT**		*p*-value
	**HYPOPERFUSED** (n = 12)	**OTHER** (n = 3)	
Age at MRI in y, median (IQR)	5.8 (0.9 to 7.8)	8 (5 to 10)	0.46
Age at epilepsy onset in y, median (IQR)	8.2 (2.4 to 13.5)	11.4 (6.9 to 13.2)	0.56
Epilepsy duration in y, median (IQR)	1.5 (0.4 to 4.4)	3 (1.7 to 3.2)	0.89
Seizure frequency ≥30 monthly, n (%)	5 (42%)	3 (100%)	0.24
Presence of focal slowing, n (%)	6 (50%)	2 (67%)	1
Presence of spikes, n (%)	5 (50%)	3 (100%)	0.51
Presence of frequent spikes, n (%)	0 (0%)	2 (67%)	0.12
LEAT volume in mm^3, median (IQR)	10404 (1977 to 11834)	18268 (4614 to 27899)	0.31
Deep seated lesion, n (%)	4 (33%)	3 (100%)	0.16

IQR: inter-quartile range; LEAT: low-grade epilepsy associated tumors; y: years; $: Mann-Whitney test

Supplementary Table 3. Perfusion changes in arterial spin labelling (ASL) larger than the low-grade epilepsy associated tumors (LEAT) did not correlate with clinical, EEG, or MRI findings.

The extent of perfusion changes in the ASL is provided in relation to clinical features, EEG, and anatomical MRI findings.

**CLINICAL, EEG, AND MRI FEATURES**	**ASL PERFUSION EXTENT**		*p*-value
	**LARGER** (n = 5)	**EQUAL** (n = 8)	
Age at MRI in y, median (IQR)	2.5 (2.1 to 10.8)	8.2 (4.7 to 13.7)	0.46
Age at epilepsy onset in y, median (IQR)	2.0 (0.6 to 6.0)	6.5 (1.8 to 11.0)	0.12
Epilepsy duration in y, median (IQR)	2.0 (1.2 to 4.8)	0.8 (1.2 to 4.8)	0.48
Seizure frequency ≥30 monthly, n (%)	1 (20%)	5 (63%)	0.36
Presence of focal slowing, n (%)	3 (60%)	4 (50%)	0.20
Presence of spikes, n (%)	2 (40%)	5 (63%)	0.83
Presence of frequent spikes, n (%)	0 (0%)	1 (17%)	1
LEAT volume in mm^3, median (IQR)	10404 (1977 to 11834)	18268 (4614 to 27899)	0.31
Deep seated lesion, n (%)	3 (38%)	2 (40%)	1

IQR: inter-quartile range; LEAT: low-grade epilepsy associated tumors; y: years; $: Mann-Whitney test

Bibliography

1. Louis DN, Perry A, Wesseling P, Brat DJ, Cree IA, Figarella-Branger D, et al. The 2021 WHO Classification of Tumors of the Central Nervous System: a summary. Neuro Oncol. 2021; 23(8):1231–51.

2. Luyken C, Blümcke I, Fimmers R, Urbach H, Elger CE, Wiestler OD, et al. The Spectrum of Long-term Epilepsy–associated Tumors: Long-term Seizure and Tumor Outcome and Neurosurgical Aspects. Epilepsia. 2003; 44(6):822–30.

3. Slegers RJ, Blumcke I. Low-grade developmental and epilepsy associated brain tumors: a critical update 2020. acta neuropathol commun. 2020; 8(1):27.

4. Berry RB, Brooks R, Gamaldo C, Harding SM, Lloyd RM, Quan SF, et al. AASM Scoring Manual Updates for 2017 (Version 2.4). Journal of Clinical Sleep Medicine. 2017; 13(05):665–6.

5. Tortora D, Cataldi M, Severino M, Consales A, Pacetti M, Parodi C, et al. Comparison of Qualitative and Quantitative Analyses of MR-Arterial Spin Labeling Perfusion Data for the Assessment of Pediatric Patients with Focal Epilepsies. Diagnostics. 2022; 12(4):811.

6. Gennari AG, Bicciato G, Lo Biundo SP, Kottke R, Stefanos-Yakoub I, Cserpan D, et al. Lesion volume and spike frequency on EEG impact perfusion values in focal cortical dysplasia: a pediatric arterial spin labeling study. Sci Rep. 2024; 14(1):7601.
